# End user experience of a widely used artificial intelligence based sepsis system

**DOI:** 10.1093/jamiaopen/ooae096

**Published:** 2024-10-07

**Authors:** Ayomide Owoyemi, Ebere Okpara, Megan Salwei, Andrew Boyd

**Affiliations:** Department of Biomedical and Health Informatics, University of Illinois at Chicago, Chicago, IL 60612, United States; Department of Pharmacy Systems, Outcomes and Policy, University of Illinois at Chicago, Chicago, IL 60612, United States; Department of Anesthesiology, Vanderbilt University Medical Center, Nashville, TN 37232, United States; Department of Biomedical Informatics, Vanderbilt University Medical Center, Nashville, TN 37232, United States; Department of Biomedical and Health Informatics, University of Illinois at Chicago, Chicago, IL 60612, United States

**Keywords:** workflow, usability, sepsis, electronic health records, clinical setting

## Abstract

**Objectives:**

Research on the Epic Sepsis System (ESS) has predominantly focused on technical accuracy, neglecting the user experience of healthcare professionals. Understanding these experiences is crucial for the design of Artificial Intelligence (AI) systems in clinical settings. This study aims to explore the socio-technical dynamics affecting ESS adoption and use, based on user perceptions and experiences.

**Materials and Methods:**

Resident doctors and nurses with recent ESS interaction were interviewed using purposive sampling until data saturation. A content analysis was conducted using Dedoose software, with codes generated from Sittig and Singh’s and Salwei and Carayon’s frameworks, supplemented by inductive coding for emerging themes.

**Results:**

Interviews with 10 healthcare providers revealed mixed but generally positive or neutral perceptions of the ESS. Key discussion points included its workflow integration and usability. Findings were organized into 2 main domains: workflow fit, and usability and utility, highlighting the system’s seamless electronic health record integration and identifying design gaps.

**Discussion:**

This study offers insights into clinicians’ experiences with the ESS, emphasizing the socio-technical factors that influence its adoption and effective use. The positive reception was tempered by identified design issues, with clinician perceptions varying by their professional experience and frequency of ESS interaction.

**Conclusion:**

The findings highlight the need for ongoing ESS refinement, emphasizing a balance between technological advancement and clinical practicality. This research contributes to the understanding of AI system adoption in healthcare, suggesting improvements for future clinical AI tools.

## Introduction

### Background

Artificial Intelligence (AI) has proven beneficial in harnessing and utilizing the mass of data created in the healthcare setting using statistical modeling, and computing power to draw insights and support care delivery. It is transforming the way that data is utilized and comprehended in the clinical context, offering insightful information, and enhancing the efficiency of patient care.^1^ By employing sophisticated algorithms, AI technologies are positioned to revolutionize disease diagnostic, treatment personalization, and patient outcome prediction, using the wealth of data generated within healthcare settings.^2^^,^^3^ This demonstrates AI’s extensive potential to transform healthcare from augmenting diagnostic accuracy to optimizing treatment pathways and improving operational efficiencies.^3–5^ One of the most significant advantages of AI is its ability to digest and interpret complex and voluminous datasets, which have traditionally been beyond human capacity to analyze effectively.^5^ These capabilities facilitate a deeper understanding of patient health, enabling more precise and timely interventions.

Sepsis management is one of such areas where AI shows great potential.^6^^,^^7^ Sepsis is a dysregulated and life-threatening host response to infection which involves a complex chain of systemic inflammation, circulatory abnormalities, and potential multi-organ failure.^8^^,^^9^ Sepsis affects over 1.7 million United States adults annually, resulting in about 350 000 deaths; it incurs the highest management costs of all hospital admissions in the United States.^10^^,^^11^ Early detection and timely administration of antibiotics, and other care regimens protocols are crucial for improving outcomes in sepsis patients.^9^ The gold standard test, blood culture, has an unacceptably long turnaround time and low sensitivity when it comes to managing sepsis, where prompt detection and diagnosis are essential for effective treatment.^12^ To address this, computerized early warning tools emerged to support clinicians in sepsis detection and clinical decision-making.^13^ These early sepsis screening tools varied from paper-based algorithms to independent applications used in specific units like the intensive care units (ICUs) to guide patient assessments.^14^ However, as AI capabilities advanced, more sophisticated clinical decision support systems integrating sepsis prediction models into Electronic Health Records (EHRs) were developed.

The urgent demand for prompt and precise sepsis diagnostics has catalyzed the emergence of AI as an essential tool, for detecting early sepsis indicators and enabling swifter interventions.^7^ One of such AI tools is the Epic Sepsis System (ESS), a clinical decision support tool integrated into the widely used Epic EHR system.^15^ The ESS has been in use at the University of Illinois Health (UI Health) for over 2 years. UI Health presently uses version 1 of the Sepsis system. Epic Systems Corporation, one of the largest EHR vendors in the world, developed the ESS, which has seen wide adoption across hundreds of hospitals in the United States. It uses a penalized logistic regression model trained on a large dataset of about 405 000 adult patient interactions spanning 3 health care systems between 2013 and 2015, to detect patterns of vital signs, laboratory results, and other variables associated with suspected sepsis.^16^ When a patient meets certain criteria based on the model’s predictions, the ESS triggers a real-time sepsis alert visible to clinicians in the EHR interface. The ESS model showed good performance during validation, with an area under the receiver operating curve (AUROC) of 0.76-0.88 for predicting sepsis across multiple healthcare facilities in Colorado, however another validation conducted at another facility in showed AUROC of 0.63.^16^^,^^17^ However, little is known about the human experience with using this system for sepsis detection; it is imperative to understand the end-user experience of systems like the ESS.

### Significance

Most studies evaluating the ESS have focused on the technical accuracy and efficacy of the system, leaving a gap in our understanding of how they are experienced and perceived by healthcare professionals in real-world settings.^15^^,^^17^ As this is one of the most widely used AI systems in clinical care, having an insight into these aspects will. offer significant lessons for other AI systems being designed for the clinical setting.^18^ Knowing the environment in which the technology will be used, how it will integrate with workflows and support delivery of clinical tasks, and how users will react to it are all crucial. Salwei and Carayon proposed a sociotechnical framework for AI integration which emphasized three key considerations: the integration of AI with the healthcare work system (including system elements, people, tasks, tools, environment, and organization), its incorporation into the clinical workflow’s temporal aspects, and its support for decision-making processes while fostering transparency and clinician trust in the technology.^19^ According to a National Academy of Medicine report, there is a need to “understand the technical, cognitive, social, and political factors in play and incentives impacting integration of AI into health care workflows.”^20^ The aim of this study is to understand the socio-technical dynamics affecting the use and adoption of the ESS based on the experience and perception of its end users.

## Materials and methods

This study took place between September and November 2023. Ethical approval for this study was granted by the University of Illinois Health Institutional Review Board (IRB: STUDY2023-0535-MOD003).

### Recruitment

A qualitative approach was used to understand the experience and perception of clinicians who used and interacted with the ESS at the UI Health Systems in Chicago. We recruited Resident Doctors and Nurses who interacted with the ESS at least once in the last 1 month for in-depth interviews. Participants were selected through a purposive sampling method. Participants were recruited through email outreaches to a contact list of staff at the facility, emails from supervisors and listservs. Participants who were willing to give their consent and had experience using ESS tools in UI health in a clinical setting (eg, Doctors, Nurses) were considered eligible. To achieve a diverse perspective on the use of ESS in UI health, we did not exclude participants based on qualifications, professional grade, or demographics.

### Data collection

One researcher (A.O) conducted each interview via Zoom. Interviews lasted on average 25 minutes. We concluded data collection when saturation was reached. Saturation was calculated using the stopping criterion as outlined by Francis et al.^21^ The process involves conducting a prior number of interviews (eg, interviewing to discover new codes, after which a predetermined number of interviews are planned to be conducted after there are no more new codes being generated). The interview session was recorded after the collection of consent from the participants. An interview guide was based on a slightly modified version of the interview guide previously used by Salwei et al to investigate Clinical Decision Support and workflow integration in another study.^19^ One (A.O) researcher manually transcribed the interviews; no personally identifiable information was gathered in the transcribed data.

### Data analysis

A codebook was created to outline the objectives of the coding, coding framework, and definitions of the codes. To ensure validity, Two researchers (A.O and E.O) collaboratively created the codebook and framework, validating the codes through iterative discussion. A third researcher (M.S) reviewed and finalized the codebook. To analyze the data, we adopted the content analysis approach which is a systematic and objective method to describe and quantify a topic by making valid inferences from data.^22^ We used a deductive and inductive content analysis approach following the process outlined by Elo and Kyngas.^23^ This process involved three major phases of preparation, organizing, and reporting. The initial codes were deductively generated based on the frameworks by Sittig and Singh, and Salwei and Carayon.^24^^,^^25^ We inductively coded topics that arose that did not fit into the existing coding framework.^26^ All coding and analysis were conducted in Dedoose, a qualitative and mixed methods data analysis web-based software that is used for organizing, coding, annotating, and visualizing data.^27^ Following the conclusion of the coding of all interview transcripts, the generated codes were reviewed by two other researchers (E.O and M.S) to ensure consistency across the transcripts. These codes were then reviewed and categorized based on two broad domains from a synthesis of the two frameworks used.

## Result

### Description of participants

We interviewed a total of 10 healthcare-providers: five of them were Resident Doctors and the other five participants were Nurses. Their clinical work experience ranged from three months to 29 years. Their frequency of interaction with the ESS ranged from once a month to as much as 10 times in a single day (on average).

### Overall perception of the ESS

Overall sentiments of clinicians were mixed, with an inclination mostly towards positive and neutral perceptions. Some participants (Table 1), especially those with fewer years of practice, appreciated the system’s interface, finding it visually appealing and user-friendly. They highlighted the ease of use and the system’s effectiveness in immediately drawing attention to potential sepsis cases. The color highlights were seen as beneficial for quickly identifying critical issues, and the ability to order labs directly from the system was praised for its convenience. Conversely, more experienced clinicians, particularly those interacting with the system frequently (Table 2), expressed negative sentiments, noting its intrusiveness and disruption to workflow. Concerns were raised about the system’s sensitivity, leading to frequent, sometimes unnecessary alerts. The lack of specific information about why alerts were triggered and difficulties in retrieving past alert information were major drawbacks. Overall, more Nurses had a positive perception of the tool than Doctors (Table 3).

**Table 1.** Perception of the ESS by years of practice.

	Positive	Neutral	Negative
2 years and below	4	1	1
Greater than 2 years	2	1	1

**Table 2.** Perception of the ESS by frequency of interaction.

	Positive	Neutral	Negative
Infrequently (once daily or less)	5	0	0
Frequently (multiple times daily or more)	1	2	2

**Table 3.** Perception of the ESS by role.

	Positive	Neutral	Negative
Nurse	4	1	0
Doctor	2	1	2

Participants discussed their workflow with the ESS, its integration into their work environment, and the system’s usability and utility, focusing on ease of use and functionality. The factors that came up were categorized under 2 main domains which are workflow fit, and usability and utility (Supplementary Table).

### Workflow fit

Clinicians found the integration of the ESS with the EHR to be a smooth process that made their workflow more efficient. However, they encountered difficulties when it came to manually reviewing patient charts within the ESS. One Doctor mentioned they routinely conduct an independent review of the alerts before responding to ensure accuracy. In the ICU, the ESS led to increased stress levels among staff due to the constant stream of alerts. While the system was found to be counterproductive in post-operative care, due to predictable sepsis alerts, it proved beneficial for managing new patient admissions and facilitating direct lab orders.

The frequency of alerts presented a challenge, particularly during times of high patient volume, leading clinicians to sometimes prioritize other tasks over responding to the ESS. This aspect of the system impacted how Nurses and Doctors communicated and made decisions in response to alerts. There was a preference among clinicians to receive alerts at their workstations instead of in-patient rooms to reduce the number of stimuli and ensure that physical assessments of patients could be conducted effectively after an alert was received. Furthermore, receiving alerts on mobile units, such as Computers on Wheels (COWs), was found to be particularly disruptive, especially during emergency situations.

### Usability

Clinicians appreciated the way the ESS presented information, highlighting the benefits of consolidated data and the use of color coding. However, they pointed out the system’s shortcomings, such as the absence of trend data and a layout that felt overcrowded. There was notable frustration regarding the user interface, especially the inability to reopen the alert screen once it had been closed. This limitation, along with the system’s failure to clearly explain the reasons behind alerts, significantly hindered effective communication between Nurses and Doctors. One Nurse shared a practical example of how this lack of clarity affected their ability to discuss patient care with doctors.

Furthermore, the clinicians found the range of actions suggested by the ESS in response to alerts to be too limited. The need for a broader spectrum of actionable responses was highlighted, indicating a gap in the system’s design. Despite these challenges, the ESS was acknowledged as a valuable tool in patient care, particularly for its role in drawing immediate attention to critical issues. While the alert feature was sometimes seen as intrusive, its overall contribution to ensuring prompt attention to urgent patient needs was well-regarded.

### Reasons for dismissing the alert

Clinicians often bypassed the ESS alert when it conflicted with their clinical assessment or when the patient was already under sepsis evaluation. Another reason stated for dismissing the alert was its intrusion into urgent clinical tasks, especially when immediate patient care is essential. Clinicians also dismiss the alarm when they are not part of the primary care team or when the patient is already under sepsis evaluation, as they add no new information.

## Discussion

This study explored the experiences of clinicians using the ESS integrated into the Epic EHR. This study provides insights into the socio-technical factors influencing the adoption and effective use of such systems. The interviewed clinicians had different frequencies of interaction with the ESS, from infrequent to multiple times daily. Overall, the ESS was praised for its seamless integration with the EHR. However, challenges were noted, such as the inability to conduct manual review of patients within the ESS window without having to exit the alert. This was important to clinicians because they wanted to conduct their own clinical evaluation before making a final decision or following the suggested actions by the ESS. Similar findings were seen in a study by Silvestri et al which showed that Doctors preferred to make final decisions off their own assessments and preferred being presented with relevant information to do that.^28^ This highlights the significance of addressing specific user needs to improve satisfaction with clinical decision support tools.^29^ Clinicians might prefer an approach where the tool is serving an augmentation role where it offers an alternative source of information to help them reach conclusions rather than where it tells them what to do.^30^

Clinicians also highlighted a gap in the system’s design: the inability to re-access alerts after exiting them. This issue is at odds with the workflow integration model proposed by Salwei, which emphasizes the importance of allowing users to interrupt and later return to tasks.^19^ Another issue clinicians had with the ESS was the added stress from constant alerts especially in high-alert settings like ICUs and post-operative care. This is similar to findings from a systematic review on AI based CDSS conducted by Wang et al which showed workflow misalignment as a challenge.^29^ Alert fatigue has been identified as one of the biggest challenges in implementing and adopting Best Practice Alert systems in any healthcare setting.^28^^,^^29^

The system’s impact on workflow varied, with frequent alerts being disruptive under heavy patient loads, leading to prioritization of tasks based on urgency, this was not the case in another study which showed the alert to specially trained Nurses who then notified Doctors of the alerts thereby reducing the pressure on clinicians in directly dealing with the alerts.^31^ Another significant challenge encountered was the lack of clarity regarding the workings of the ESS, particularly concerning the reasons behind triggered alerts and the underlying factors. This transparency has been identified as crucial for building clinician trust in the system.^30^

The study revealed mixed perceptions of the ESS’s usability and utility. Clinicians appreciated the system’s consolidated data presentation and color coding but criticized its crowded layout and lack of trend data. A notable issue identified was the system’s design flaw that prevented users from revisiting alert screens once they were closed, along with vague alert explanations. This flaw hindered effective communication between Nurses and Resident Doctors, which has been found in other studies.^31^ For instance, Sandhu et al found that Nurses faced challenges in discussing alert details with Doctors due to their inability to reference or explain the specific factors triggering the alerts.^31^

The overall perception of the ESS among clinicians was influenced by their years of practice and how frequently they interacted with the system. More experienced clinicians tended to have less positive views, possibly because they perceived a lower utility in the system, believing in their own proficiency to identify sepsis—a finding echoed in the study by Sandhu et al.^31^ Overall, most of the clinicians in the study had a positive perspective of the ESS which is similar findings from the Wang et al study.^29^ However, perceptions of the system varied by role, with Nurses generally viewing it more favorably than Doctors. This discrepancy may stem from the differing expectations and roles of Nurses and Doctors, as well as their respective views on the ESS contribution to their workflow.^32^

## Implication

Several socio-technical barriers to implementing ESS tools in healthcare were identified in this study. Ongoing monitoring and evaluation of ESS implementation, through metrices such as usage measurements, clinician feedback, and patient outcomes, are required to enhance the system and assess its real-world impact. Going forward, ESS integration efforts must prioritize human-centered design, and the interface should be tailored to fit clinical workflows seamlessly. The alerts in addition to being clear, should be comprehensible, enabling clinicians to understand the rationale behind ESS prompts and support collaborative decision-making across care teams. Furthermore, regular training and change management initiatives are critical to ensure clinicians are well-versed in ESS features and can effectively incorporate the system into their practice.

## Strength and limitations

A key strength of this study is that we interviewed clinical professional with lived experiences in the use of ESS. By including both Doctors and Nurses with varying levels of clinical experience, ranging from three months to 29 years, and with different frequencies of interaction with the ESS, this study captured a range of perspectives and lived experiences. This sample size and the variety in the sample allowed for saturation and provided insightful understanding of how ESS fits in the context of the healthcare setting studied. The qualitative approach, through in-depth interviews, allowed for the exploration of the factors influencing the uptake and acceptance of such AI tools by end-users. The risk of researcher bias is mitigated in our study through a collaborative approach, involving two researchers in the development of a detailed codebook and coding process, ensuring coding validity. The study findings are offering valuable insights into strategies that may improve the acceptability and effective implementation of AI systems in clinical practice.

One potential limitation of the study is that it focuses on a specific tool for a specific pathology (Sepsis), however, the findings are more generalizable as this is a widely used tool across health systems in the United States. Another limitation is the study scope which excluded non-clinical staff members and patients. While the aim of this study, was to capture the perspectives of direct clinical end-users, capturing the experiences of other stakeholders (administrative or technical support staff), could provide additional insights and enhance the generalizability of the findings. Additionally, while clinicians are the primary end-users of the ESS studied, the implementation of such AI-enabled clinical decision support systems can potentially impact patient-provider interactions and communication, an aspect that warrants further investigation. This study was conducted within a single healthcare system, therefore, the results might not extend to other settings with varying workflows. Replicating the study across multiple sites and diverse contexts would strengthen the external validity of the results.

## Conclusion

These findings suggest the need for ongoing refinement of the ESS, emphasizing the balance between technological efficiency and clinical practicality. This study can inform efforts to effectively implement Clinical Decision Support tools like the ESS in real-world healthcare settings by addressing the socio-technical factors influencing their use and acceptance among clinicians.

## Supplementary Material

ooae096_Supplementary_Data

## Data Availability

The data underlying this article will be shared on reasonable request to the corresponding author.
