# Supplementary material for: End user experience of a widely used artificial intelligence based sepsis system
Source: JAMIA Open. 2024 Oct 7;7(4):ooae096. doi: 10.1093/jamiaopen/ooae096 (PMC11458550; doi:10.1093/jamiaopen/ooae096)
Supplement: ooae096_Supplementary_Data [file ooae096_supplementary_data.docx]

**Supplementary Table. Factors affecting perception and experience with selected quotes.**

| **DOMAIN** | **FACTORS** | **DESCRIPTION** | **EXAMPLE QUOTES** |
| --- | --- | --- | --- |
| **WORKFLOW FIT** | Integration with the Electronic Health Record (EHR) | Positive perception of its level of integration | ***"I can't remember the last time that I ordered like the lactic acid with the two-hour reflex or the blood cultures just directly from the order set, without first kind of trying to figure out what exactly would be going on, what exactly would trigger the alert and what changes may have occurred."*** |
|  | Clinical Task Impact | Unhelpful for ICU and post-operative care | *"****I expect nearly all of my post-op patients to trigger a sepsis alert…and so, if I'm getting an alert on something I expect constantly every time I open the chart, then it becomes an inconvenience and annoyance that it's telling me like, I know, I know, but now I have to click an extra step. And if I don't click the right thing, I'm ordering blood cultures on someone I don't wanna necessarily order blood cultures on."*** |
|  | Timing and Patient Burden Impact | Timing of alert was disruptive in some cases | ***"…we have an hour to send the labs in anyway. So if I'm in the middle of something more important or more of a higher priority, I will do that first. But if I'm in the middle of doing like, let's say just charting or something that's not less, less of a priority or that is less of a priority, I will do this instead, cos we only have an hour to do that labs."*** |
|  | Clinician Interaction Impact | The ESS influenced communication between Nurses and Resident Doctors. | ***“… pops up for the Nurses before I can even see it. So, they'll come to me and say, hey, they triggered a sepsis alert. Do you want us to follow the protocol? Which is like grab blood cultures in some other labs and such. I'll be like either yes or no. Yeah.”*** |
|  | Environmental Impact | Clinicians preferred receiving alerts at workstations. | *"****It's better if it pops up at the workstation because like, there's so many bells and whistles already in the patient rooms and it would just be less overwhelming for both staff and the family and patient if there's just less like stimuli altogether."*** |
|  | Patient Impact | Frequent alerts for specific patients led to clinician frustration. | ***"So, I work with neurosurgery patients. So, all my patients are over the age of 18 adults and have some type of neurological injury or problem and oftentimes are ventilated with a machine and just being on a ventilator in itself sometimes with like a temperature of 99 it'll trigger the alert."*** |
|  | Technological Impact | Alerts on mobile units were seen as disruptive | ***“it doesn't play a role in when using the mobile device, thankfully. Because that would just make it really... Because on the mobile, you know, you're trying to do just quick stuff while you're walking around and things, and responding to messages. So yeah, it definitely wouldn't fit in that sense."*** |
| **USABILITY & UTILITY** | Mode of Presentation | Issues with lack of trend data and crowded layout. | ***"I don't like how it's, how I'm not able to read through it because it's very crowded with a lot of information and it's not properly laid out. So, I can't read why they're calling the sepsis alert and I can't focus on what they're saying."*** |
|  | Ease of Interaction and Retrieval | Frustration over the inability to revisit the alert screen once closed. | ***"You know, I don't like the fact that when you click out of it, it's gone and you have no idea, unless you wrote it down or something, you have no idea really what was the cause of the sepsis alert…*** ***if I didn't notice that when I clicked out of it…it would be kind of hard for me. If I could kind of go back and kind of check maybe the vitals, check the labs and see maybe it was that probably, you know.”*** |
|  | Relevance of Information | Lack of clarity in explaining the reasons for alerts. | ***"What I don't like about it though is it doesn't tell you specifically why the sepsis alert was triggered. That's the first thing the Doctors always ask us when we tell them, well, why did they trigger a sepsis alert?...So, like that last one, the patient's temperature was 113. So of course, that would be a reason to trigger a sepsis alert, but it's also not specifically saying this is why the sepsis alert was triggered."*** |
|  | Gaps in Suggested Actions | Limited action options available. | ***"But if we want more...in our thought process when we see a patient that we're concerned about sepsis would be to order further investigation studies to find a source. And I don't necessarily see that."*** |
|  | Appropriateness of Action Buttons | Action buttons useful to the tasks required | ***"I do like that you could order your lab directly from the screen. Cause that speeds up the process."*** |
|  | Effectiveness of Alert | Useful in calling attention to sepsis. | ***"I do think that the fact that it comes up like big takes up the whole screen ultimately is a good thing, good for the interface because it prompts you to like address it right away."*** |
